# Supplementary material for: Free Sugars Intake, Sources and Determinants of High Consumption among Australian 2-Year-Olds in the SMILE Cohort
Source: Nutrients. 2019 Jan 13;11(1):161. doi: 10.3390/nu11010161 (PMC6356352; doi:10.3390/nu11010161)
Supplement: Supplementary file 1 [file nutrients-11-00161-s001.pdf]

Table S1. Characteristics of participants who completed both the 1 and 2 year dietary questionnaires.

	**Completed both questionnaires (*n* = 682)**			**Did not complete both questionnaires (*n* = 1499)**			p^a
	**n**	**mean**	**SD**	**n**	**mean**	**SD**	
Mother's age at birth (years)	680	30.9	4.8	1421	29.4	5.6	<0.001
Total number of children	661	1.7	0.9	1206	1.9	1.0	0.005
Child's birthweight (g)	674	3411	562	1409	3329	575	0.002
Child's age at 24HR (months)	682	13.1	1.0	146	13.1	0.9	0.736
Child's age at FFQ (months)	670	25.2	1.1	451	26.1	2.0	<0.001
	**n**	%		**n**	%		
Mother's age at birth (years)							<0.001
<25	55	8.1		281	19.8		
25-34.99	481	70.7		872	61.4		
≥35	144	21.2		268	18.9		
Mother's pre-pregnancy BMI (kg/m^2)							0.002
<25	398	61.8		688	53.4		
25-29.99 (overweight)	132	20.5		323	25.1		
≥30 (obese)	114	17.7		278	21.6		
Mother's Country of Birth							<0.001
Australia or New Zealand	513	75.8		940	66.2		
Other	164	24.2		480	33.8		
Mother's Education							<0.001
high school / vocational	273	40.3		863	60.5		
some university and above	405	59.7		563	39.5		
IRSAD							<0.001
Deciles 1–2 (most disadvantaged)	93	13.7		369	26.3		
Deciles 3–4	141	20.8		305	21.7		
Deciles 5–6	141	20.8		249	17.7		
Deciles 7–8	137	20.2		248	17.7		
Deciles 9–10 (most advantaged)	165	24.4		233	16.6		
Total number of children							0.082
1	320	48.4		543	45.0		
2	240	36.3		430	35.7		
≥3	101	15.3		233	19.3		
Child's sex							0.175
Male	374	54.8		772	51.7		
Female	308	45.2		721	48.3		
Mother's smoking status at 1 year							0.035
no	625	93.0		540	89.7		
yes	47	7.0		62	10.3		

[a] *p*-values for responders v non-responders using t-test (continuous) or Pearson Chi Square test (categorical).
24HR: 24-hour recall; FFQ: Food Frequency Questionnaire; BMI: Body Mass Index IRSAD: Index of Relative Socioeconomic Advantage and Disadvantage

Table S2. Participant characteristics, sub-set for sensitivity analysis: responders at 2 years with calculated EER from weight data vs reference EER imputed for age and sex.

	Calculated EER from weight (*n* = 918)			Imputed (reference) EER (*n* = 125)			*p*[a]
	n	mean	SD	n	mean	SD	
Mother's age at birth (years)	915	30.6	4.9	125	30.2	5.4	0.483
Total number of children	890	1.8	0.9	123	1.7	0.9	0.806
Child's birthweight (g)	908	3398	548	124	3355	537	0.411
Child's age at FFQ (months)	895	25.5	1.5	120	25.6	1.6	0.433
	n	%		n	%		*p*[a]
Mother's age at birth (years)							0.043
<25	86	9.4		19	15.2		
25-34.99	642	70.2		75	60.0		
≥35	187	20.4		31	24.8		
Mother's pre-pregnancy BMI (kg/m^2)							0.351
<25	493	57.1		72	64.3		
25-29.99 (overweight)	197	22.8		21	18.8		
≥30 (obese)	173	20.0		19	17.0		
Mother's Country of Birth							0.799
Australia or New Zealand	665	73.1		90	72.0		
Other	245	26.9		35	28.0		
Mother's Education							0.903
high school / vocational	396	43.4		55	44.0		
some university and above	516	56.6		70	56.0		
IRSAD							0.256
Deciles 1–2 (most disadvantaged)	144	15.8		28	22.6		
Deciles 3–4	195	21.4		19	15.3		
Deciles 5–6	183	20.1		27	21.8		
Deciles 7–8	177	19.4		22	17.7		
Deciles 9–10 (most advantaged)	213	23.4		28	22.6		
Total number of children							0.734
1	421	47.3		59	48.0		
2	322	36.2		47	38.2		
≥3	147	16.5		17	13.8		
Child's sex							0.755
Male	491	53.5		65	52.0		
Female	427	46.5		60	48.0		
Mother's smoking status at 1 year							0.135
no	767	92.9		95	88.8		
yes	59	7.1		12	11.2		

[a.] *p*-values for calculated v imputed EER using t-test (continuous) or Pearson Chi Square test (categorical). 24HR: 24-hour recall; FFQ: Food Frequency Questionnaire; BMI: Body Mass Index IRSAD: Index of Relative Socioeconomic Advantage and Disadvantage

Table S3. Free Sugars intakes at 2 years of age, sub-set for sensitivity analysis: responders with imputed EER excluded (*n* = 918).

	Median	Percentile 25th,	75th	Range	
Free Sugars (g/day)	22.0	12.7,	37.6	0.6	– 140.7
Total Sugars (g/day)	77.2	55.6,	104.7	9.4	– 294.8
Estimated Energy Requirement (kJ/day)	4841	4394,	5260	2644	– 8193
Percentage of Estimated Energy Requirement from Free Sugars (%)	7.5	4.5,	13.2	0.2	– 61.3

Table S4. Tertile of free sugars intakes from 1 to 2 years of age, sub-set for sensitivity analysis: responders with imputed EER excluded (*n* = 623).

		Free Sugars at 2 Years		
		**Low**	**Mid**	**High**
**Free Sugars at 1 year**	**Low**	104 (16.7%)	66 (10.6%)	34 (5.5%)
	**Mid**	76 (12.2%)	68 (10.9%)	68 (10.9%)
	**High**	48 (7.7%)	60 (9.6%)	99 (15.9%)

Weighted Kappa 0.221, p <.001

Table S5: Participant characteristics associated with high consumption of free sugars at 2 years, sub-set for sensitivity analysis: responders with imputed EER excluded (n = 829).

	Free sugars highest tertile		Free Sugars Intake ≥10%EER	
	**PR**	**95%CI**	**PR**	**95%CI**
Mother's age at birth (years)				
<25	1.59	[1.10, 2.30]	1.51	[1.09, 2.11]
25-34.99	1.11	[0.84, 1.44]	1.09	[0.86, 1.38]
≥35	1.00		1.00	
Mother's pre-pregnancy BMI (kg/m^2)				
<25 Healthy weight or below	1.00		1.00	
25-29.99 Overweight	1.08	[0.86, 1.38]	1.02	[0.82, 1.26]
≥30 Obese	0.98	[0.75, 1.28]	0.92	[0.71, 1.19]
Mother's Country of Birth				
Other	1.47	[1.18, 1.84]	1.54	[1.26, 1.88]
Australia, New Zealand	1.00		1.00	
Mother's Education				
University	0.84	[0.68, 1.04]	0.83	[0.68, 1.01]
High school / vocational	1.00		1.00	
IRSAD				
Deciles 1–2 (most disadvantaged)	1.49	[1.10, 2.02]	1.40	[1.06, 1.84]
Deciles 3–4	1.32	[0.99, 1.78]	1.34	[1.04, 1.73]
Deciles 5–6	0.95	[0.68, 1.33]	0.93	[0.69, 1.26]
Deciles 7–8	0.99	[0.71, 1.37]	0.98	[0.73, 1.31]
Deciles 9–10 (most advantaged)	1.00		1.00	
Total number of children				
1	1.00		1.00	
2	1.31	[1.05, 1.64]	1.20	[0.98, 1.47]
≥3	1.42	[1.07, 1.88]	1.33	[1.03, 1.72]
Child's sex				
Female	0.90	[0.74, 1.10]	0.94	[0.79, 1.12]
Male	1.00		1.00	

PR: Prevalence Ratio; BMI: Body Mass Index (kg/m2); IRSAD: Index of Relative Socioeconomic Advantage and Disadvantage.
